# Development and clinical testing of individual immunoassays for the quantification of serum glycoproteins to diagnose prostate cancer

**DOI:** 10.1371/journal.pone.0181557

**Published:** 2017-08-02

**Authors:** Kathrin Endt, Jens Goepfert, Aurelius Omlin, Alcibiade Athanasiou, Pierre Tennstedt, Anna Guenther, Maurizio Rainisio, Daniel S. Engeler, Thomas Steuber, Silke Gillessen, Thomas Joos, Ralph Schiess

**Affiliations:** 1 ProteoMediX Inc., Schlieren, Switzerland; 2 NMI Tübingen, Reutlingen, Germany; 3 Cantonal Hospital St. Gallen, St. Gallen, Switzerland; 4 Martini-Klinik, Hamburg-Eppendorf, Germany; 5 Abanovus LLC, Sanremo, Italy; Duke University School of Medicine, UNITED STATES

## Abstract

Prostate Cancer (PCa) diagnosis is currently hampered by the high false-positive rate of PSA evaluations, which consequently may lead to overtreatment. Non-invasive methods with increased specificity and sensitivity are needed to improve diagnosis of significant PCa. We developed and technically validated four individual immunoassays for cathepsin D (CTSD), intercellular adhesion molecule 1 (ICAM1), olfactomedin 4 (OLFM4), and thrombospondin 1 (THBS1). These glycoproteins, previously identified by mass spectrometry using a Pten mouse model, were measured in clinical serum samples for testing the capability of discriminating PCa positive and negative samples. The development yielded 4 individual immunoassays with inter and intra-variability (CV) <15% and linearity on dilution of the analytes. In serum, *ex vivo* protein stability (<15% loss of analyte) was achieved for a duration of at least 24 hours at room temperature and 2 days at 4°C. The measurement of 359 serum samples from PCa positive (n = 167) and negative (n = 192) patients with elevated PSA (2–10 ng/ml) revealed a significantly improved accuracy (*P* <0.001) when two of the glycoproteins (CTSD and THBS1) were combined with %fPSA and age (AUC = 0.8109; *P* <0.0001; 95% CI = 0.7673–0.8545). Conclusively, the use of CTSD and THBS1 together with commonly used parameters for PCa diagnosis such as %fPSA and age has the potential to improve the diagnosis of PCa.

## Introduction

PCa is the most frequently diagnosed cancer in men and the second leading cause of male cancer related deaths in the US [1]. PSA is commonly used to diagnose PCa but owing to its low specificity, can lead to false positive results and consequently to a high number of prostate biopsies with negative diagnostic findings. Further, these unnecessary biopsies can have potential side effects [2,3]. Recent recommendations against widespread screening of men for PCa using PSA [4] have resulted in fewer men being screened for PCa, and fewer early-stage cases being detected [5].

New diagnostic tools, ideally non-invasive ones, are urgently needed to improve PCa diagnosis and reduce overtreatment. More accurate diagnostics from easily accessible sample types like blood will allow physicians and patients to make more informed decisions about potential cases of PCa and whether a prostate biopsy is required.

We have previously identified protein biomarkers for PCa diagnosis using a two-staged genetics-guided discovery approach focusing on the PI3K/PTEN cancer pathway [6]. In the first stage, glycoproteins were identified from sera and prostate tissue of wild-type and Pten-null cancer mouse models. Following prioritization of the proteins, proteomic profiles were identified in the sera of PCa patients and control individuals in a second stage validation step.

Here, our objective was to develop and validate immunoassays for the individual protein biomarkers. The transition from mass spectrometry to immunoassay technology represents an important step to enable high-throughput clinical validation in large sample cohorts. Furthermore, it facilitates clinical application of the test in routine diagnostic laboratories. The microparticle-based Luminex platform was chosen due to the improved kinetics resulting from a higher surface area of microparticles when compared to microtiter plates or tubes [7]. In addition, magnetic microparticle-based assays are more amenable to automation than tube- or microtiter plate-based assays [8] and thus widely distributed in random-access immunoassay systems. Finally, the multiplexing capabilities of the Luminex system facilitated the antibody selection process [9] for immunoassay development. It is important to note that the Luminex assays developed were used in singleplex format for the measurement of clinical samples.

In this report, we describe the development and technical validation according to the guidelines from the Center for Drug Evaluation and Research (CDER) [10] of four individual immunoassays for cathepsin D (CTSD), intercellular adhesion molecule 1 (ICAM1), olfactomedin 4 (OLFM4), and thrombospondin 1 (THBS1). These immunoassays were then clinically tested in 359 serum samples of patients diagnosed with (n = 167) and without (n = 192) PCa. All men had an elevated PSA in the range of 2–10 ng/ml. This range of PSA values is often referred to as the “diagnostic grey zone”, because many men have benign conditions, resulting in higher PSA values, and therefore, large number of false positive cases. Today if PSA is at such an intermediate level, urologists might use percent free PSA (%fPSA) as an additional aid to decide if a biopsy is necessary [11]. Our objective was to test if the additional measurement of four glycoproteins to PSA can improve PCa diagnosis in men with elevated PSA levels.

## Materials and methods

### Reagents and equipment

THBS1 calibrator and antibodies were purchased from R&D Systems. Calibrator was recombinant human THBS1 (3074-TH-050), the capture antibody was a mouse anti-human monoclonal (MAB3074) and the detection antibody was an affinity-purified, biotinylated goat anti-human polyclonal antibody (BAF3074). Recombinant human CTSD (residues 1–412), ICAM1 (residues 1–480) and OLFM4 (residues 1–510) were expressed and purified from cell culture supernatants of transfected HEK293 cells using either a Ni-NTA column for CTSD and ICAM1 or an ammonium sulfate precipitation for OLFM4 followed by a size exclusion approach. The capture and detection antibodies were mouse monoclonals generated against the recombinant full-length proteins of human CTSD, ICAM1 and OLFM4.

Buffers were obtained from Candor Bioscience and Roche Diagnostics. Streptavidin-phycoerythrin was obtained from Moss Inc. For assay development and technical validation purposes, blood was taken from healthy donors at Blutspende Schlieren, Switzerland and was used exclusively for research and assay development procedures and not for clinical testing. Blood samples were stored at 4°C and centrifuged at 2’000g for 10 min to separate serum and cellular fractions. The serum was aliquoted and stored at -80°C.

The Luminex MAGPIX system was used for the assay development and measurement of clinical serum samples and the Luminex FLEXMAP 3D system was used for technical assay validation. xPONENT (Luminex Corp., Vers. 4.2 and 4.1) and Masterplex QT (Hitatchi, Ver. 5) were used as the acquisition and analysis software. Fluorescently coded magnetic microparticles were obtained from Luminex Corporation. Luminex Magnetic Plate Separator was used to handle 96-well half area plates (derived from Corning) of microspheres during assay processing steps. During incubation, the plates were placed in an Eppendorf ThermoMixer C equipped with Eppendorf SmartBlock plates and ThermoTop. Liquid handling was performed with calibrated, adjustable, precision pipettes.

### Study population

In this case-control study, 359 serum samples from PCa positive (n = 167) and negative (n = 192) selected from the biobanks of the Cantonal Hospital St. Gallen, Switzerland and the Martini-Klinik, Hamburg-Eppendorf, Germany were used for testing. All samples were from men that had a PSA between 2 and 10 ng/ml. The Cantonal Hospital St. Gallen provided 128 serum samples collected between 2004 and 2009. The study was approved by the local ethics committee and all patients gave written informed consent. The blood samples were collected, stored at room temperature for 15–30 min and centrifuged at 1’500g in a serum separator tube. The serum was stored at -60°C or lower. 56 samples were originally drawn from men with known diagnosis of PCa and 72 samples from men without PCa (n = 72). 231 serum samples of PCa positive (n = 111) and negative men (n = 120) were selected from the Martini-Klinik, Prostate Cancer Center (University Medical Center Hamburg-Eppendorf). All samples were taken between 2011–2012 following written patient consent. The samples were allowed to stand at room temperature for 30 min. Blood was centrifuged at 2’000g for 10 min to separate serum and cellular fractions. The serum was stored at -80°C.

### Immunoassay development

Bead-based sandwich immunoassays were established on the Luminex system as follows: Capture antibodies were covalently conjugated to carboxylated Luminex microparticles and the detection antibodies were labeled with biotin according to standard procedures. 96-well half area microtiter plates (Corning Inc.) were blocked for a minimum of 15 min with 1x Blocking Reagent for ELISA (Roche Diagnostics). The capture antibody-coated microparticle/biotinylated detection antibody mix was added to the protein (sample or standard) diluted in main assay buffer, Low Cross Buffer (LCB; Candor Bioscience) within the 96-well plate. Following a 60 min incubation at 37°C and shaking at 650 rpm in an Eppendorf ThermoMixer C, the plates were washed with PBS/0.05% Tween20 using a magnetic plate separator (Luminex Corporation). Streptavidin-phycoerythrin conjugate (Moss Inc.) was added for 30 min, incubated with 650 rpm at 21°C in an Eppendorf ThermoMixer C. After washing, the bead conjugates were resuspended in Blocking Reagent for ELISA for analysis. Read-out was performed with either a Luminex FlexMap3D or Luminex MAGPIX instrument operated with xPONENT 4.1 or 4.2 software.

Serum total PSA (tPSA) and complexed PSA (cPSA) were analyzed using the ADVIA Centaur immunoassay system (Siemens Healthcare). Percent free PSA (%fPSA) was calculated using the measured values of tPSA and cPSA with the following formula %fPSA = (tPSA-cPSA)/tPSA*100.

### Immunoassay validation

Technical validation was performed following the guidelines from the Center for Drug Evaluation and Research (CDER) [10]. The validation process of the bead-based immunoassays for CTSD, ICAM1, OLFM4 and THBS1 included the assessment of sensitivity, linear range, precision, reproducibility, freeze/thaw stability, and stability at 4°C and room temperature.

For evaluation of the assay validation parameter, at least 20 independent assay runs were performed by four operators with no more than two assays per day and operator. The seven standard calibrator concentrations were measured in triplicates. The LoD was determined by measurement of 24 replicates of the zero calibrator in one assay [12]. For calculation of LoD the mean raw data of the zero calibrator (n = 24) and the two next calibrator points were calculated. A linear approximation between the blank and the two calibrator points was used (line fit) for determination of the concentration of the average zero calibrator + 3 SD value.

The LLoQ was defined as the lowest concentration of diluted calibrator protein at which the assay still fulfilled the acceptance criteria of intra-assay precision (CV) of <25% and accuracy within 25% of the nominal concentration of the calibrator concentration used for preparing the individual dilutions. The ULoQ was defined as the calibrator point that showed a precision (CV) of <20% and accuracy within 20% of the nominal concentration. Inter-assay precision was established to test reproducibility of the assay by comparing triplicate measurements of eleven samples per run on 22 independent runs on more than six different days, done by four operators. Intra-assay precision was determined to establish reproducibility of the assays by 18-fold measurement of 5 serum samples covering the assay range in 5 parallel measurements.

Dilutional linearity of the individual assays was assessed by measuring 5 samples in different serial dilutions. For evaluating short-term stability of the analyte, five individual human serum samples were stored for up to 48 hours at 4°C and up to 24 hours at room temperature prior to applying the samples to the assay. The effect of these additional storage conditions was evaluated and compared to a fresh aliquot of the same sample thawed prior to running the assay. Freeze-thaw (F/T) stability of the analyte was determined using five individual human serum samples. Aliquots of these samples were thawed on ice and refrozen (-80°C) up to three times. The recalculated values were compared to the concentration of an untreated sample aliquot.

### Statistical analysis

Statistical analysis was performed with SAS^®^ version 9.3 and GraphPad PRISM version 6.0. We performed comparisons with the Mann-Whitney test not assuming normal distribution of the data. *P* values <0.05 were considered significant. For the variable selection, both forward selection (stepwise selection with entry testing based on the significance of the score statistic, and removal testing based on the probability of a likelihood-ratio statistic based on conditional parameter estimates) as well as backward elimination (removal testing is based on the probability of the likelihood-ratio statistic based on conditional parameter estimates) was performed using SPSS Statistics software (version 23). Decision curve analysis was performed as described by Vickers and Elkin [13] using RStudio^®^ [version 0.99.46]) an integrated development environment for R [version 3.2.2] [14].

## Results

### Development of four immunoassays

For all biomarkers, except for THBS1, recombinant mammalian derived protein standards and highly specific monoclonal antibody pairs we developed. Commercially available reagents for THBS1 were purchased from R&D Systems. The antibody selection process was performed using the bead-based Luminex system taking advantage of the capability for multiplexing and simultaneous testing of multiple antibody sandwich pairs. Buffer compositions and antibody concentrations were optimized accordingly to yield the most optimal conditions for the individual immunoassays.

**Fig 1:**
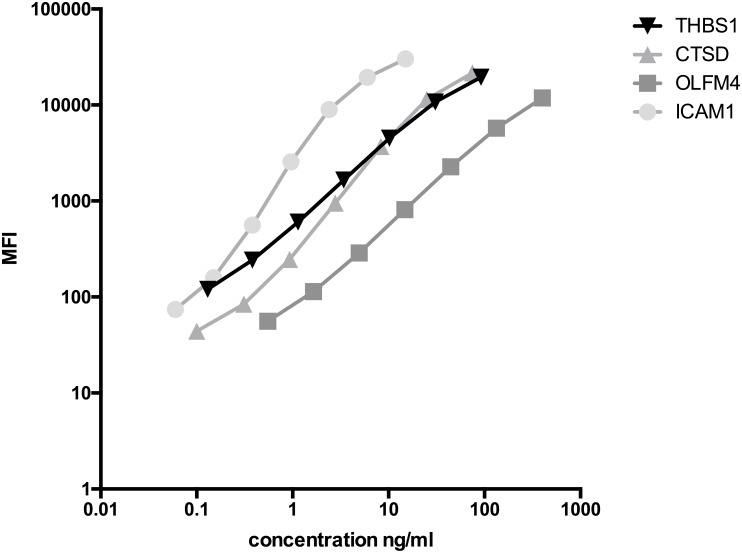
Assay calibration curves. Representative calibration curves for the CTSD, ICAM1, OLFM4, and THBS1 assays.

For the highest calibrator concentration, full-length recombinant proteins were added to the main assay buffer (LCB) and serially (S1-S7) diluted. The standard range for CTSD was 75 to 0.1 ng/ml using 3-fold dilution steps in LCB/0.5% Tween20. The highest final concentration of ICAM1 was 15 ng/ml followed by 6 serial dilutions in LCB/250 mM NaCl with a dilution factor of 2.5. Final starting concentrations for OLFM4 was 400 ng/ml followed by 1:3 dilution steps in LCB and for THBS1, starting concentration was 92 ng/ml diluted 1:3 in LCB/0.5% Tween20. Typical examples of calibration curves are shown in Fig 1. Interquartile ranges of the individual markers measured in serum samples collected from healthy donors were as follows: CTSD: 84–173 ng/ml (n = 63), ICAM1: 114–145 ng/ml (n = 43), OLFM4: 8–71 ng/ml (n = 24), and THBS1: 24’000–34’000 ng/ml (n = 40), respectively.

### Technical validation results

The LoD calculated by adding 3 SDs to the mean of 24 blanks was defined as 1.1 ng/ml for CTSD, 1.7 ng/ml for ICAM1, 2.1 ng/ml for OLFM4, and 102 ng/ml for THBS1. The LLoQ with an acceptance criterion of a CV <25% and a recovery of the nominal concentration between 75–125% was calculated to be 5.2 ng/ml for CTSD, 12.0 ng/ml for ICAM1, 14.8 ng/ml for OLFM4, and 852 ng/ml for THBS1. The ULoQ (acceptance criterion of a CV <20% and a recovery of the nominal concentration between 80–120%) was defined as 1’125 ng/ml for CTSD, 600 ng/ml for ICAM1, 3’200 ng/ml for OLFM4, and 368’000 ng/ml for THBS1.

The imprecision of the individual assays was determined by measuring multiple samples in triplicates on more than five different days, performed by two operators. Intra-assay precision (CV) for all four assays in all measured samples was <15%. The same was true for inter-assay precision, except for one out of eleven samples (19.7%) measured by OLFM4. Intra-day precision and operator to operator variability was <15% for all assays, except for OLFM4. Table 1 describes the measuring range and precision of the four immunoassays.

**Table 1:** Measuring range and precision.

Analyte	LoD, ng/mL	LLoQ, ng/mL	ULoQ, ng/mL	Inter-run CV	Intra-run CV
**CTSD**	1.1	5.2	1’125	6.2% (3.9–9.8%)	4.2% (2.8–6.1%)
**ICAM1**	1.7	12.0	600	4.2% (2.3–5.5%)	6.7% (5.6–8.3%)
**OLFM4**	2.1	14.8	3’200	13.0% (10.6–19.7%)	7.7% (3.4–14.6%)
**THBS1**	102	852	368’000	4.2% (3.3–4.8%)	10.5% (9.4–12.5%)

LoD was calculated by adding 3 SD to the mean of 24 blanks. Acceptance criterion for LLoQ and ULoQ was a CV of <25% and <20%. Inter CV was calculated by comparing triplicate measurements of 11 serum samples on 20 different runs over 5 days. Intra CV was defined by measuring 5 serum samples in 18 replicates.

Human serum samples were serially diluted and the measured concentration was recalculated considering the dilution factors. Five individual samples per analyte were measured in triplicate to consider possible matrix differences that can exist between samples and further, to check the recovery in samples with different initial analyte concentrations (S1 Fig). For all analytes, the test results were directly proportional to the concentration of analyte in the sample when diluted with sample diluent. OLFM4 concentration measured in a dilution of 1:8 was considered as the 100% reference value. For CTSD a final dilution of 1:15, for ICAM1 a dilution of 1:100 and for THBS1 a dilution of 1:4’000 was considered as the 100% reference value, respectively.

**Fig 2:**
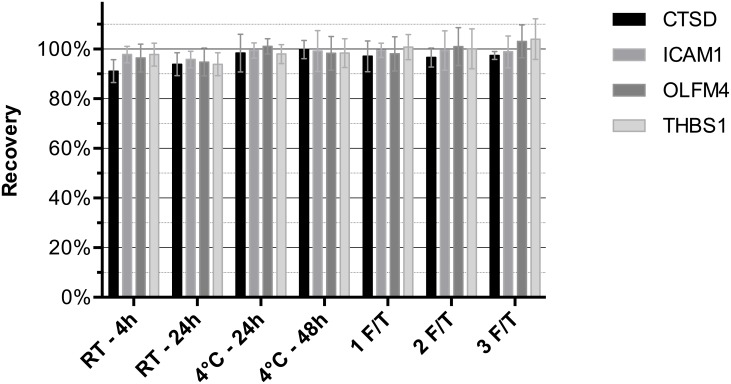
Stability experiments. Stability of CTSD, ICAM1, OLFM4, and THBS1 in serum stored at room temperature (RT), 4°C or 1 up to 3 freeze and thaw (F/T) cycles, respectively. Shown are the mean (SD; error bars) measured protein concentrations (as a percentage of the original values) in 5 different samples.

Analyte stability was tested at room temperature in 5 different serum samples. All 4 analytes were stable for at least 24 hours at room temperature. Mean values (as a percentage of the initial value for the samples) were 94% (range, 86–97%) for CTSD, 96% (92–101%) for ICAM1, 95% (85–98%) for OLFM4, and 94% (86–97%) for THBS1 (Fig 2) after 24 hours. For the same samples, stability at 4°C was tested for up to 48 hours. After 48 hours of storage, the mean measured CTSD value was 100% (96–106%) of its original value, 99% (85–106%) for ICAM1, 98% (89–104%) for OLFM4, and 98% (90–104%) for THBS1 (Fig 2). In 5 individual human serum samples subjected to 3 cycles of freezing and thawing, no change in the analyte concentration was detected. The mean measured CTSD value after the third thawing was 97% (range, 95–99%) of the original value for CTSD, 99% (90–105%) for ICAM1, 103% (94–111%) for OLFM4, and 104% (94–117%) for THBS1 (Fig 2).

### Clinical testing

359 men with PSA between 2 and 10 ng/ml undergoing prostate biopsy were included in this study. 231 samples thereof were collected at the Martini-Klinik in Hamburg, Germany and 128 originated from the Cantonal Hospital St. Gallen, Switzerland. 192 men were negative for PCa based on prostate biopsy and 167 were diagnosed with PCa (Table 2). The objective of the study was to test if a model of one or several glycoproteins together with parameters commonly used for PCa diagnosis such as PSA, %fPSA or age could potentially improve the discrimination of PCa positive and negative patients.

**Table 2:** Patient characteristics overview.

	Overall	PCa negative	PCa positive	p-value	
**Number**	359	192	167		
**Age (yrs)**	66 (61–71)	68 (61–73)	64 (59–69)	<0.001	†
**Total PSA (ng/ml)**	4.7 (3.4–6.5)	4.7 (3.2–6.3)	5.0 (3.6–6.7)	0.154	†
**Free/total PSA ratio**	0.14 (0.07–0.21)	0.18 (0.11–0.27)	0.10 (0.04–0.17)	<0.001	†
**CTSD (ng/ml)**	222 (182–266)	218 (179–261)	229 (185–279)	0.125	†
**ICAM1 (ng/ml)**	122 (102–145)	124 (105–147)	119 (100–141)	0.074	†
**OLFM4 (ng/ml)**	57 (23–123)	62 (28–112)	55 (20–55)	0.296	†
**THBS1 (ng/ml)**	23’000 (18’000–31’000)	26’000 (20’000–36’000)	21’000 (16’000–27’000)	<0.001	†

PSA = prostate specific antigen; CTSD = cathepsin D; ICAM1 = intercellular adhesion molecule 1; OLFM4 = olfactomedin 4; THBS1 = thrombospondin 1. Data are presented as median (interquartile range).

^†^ Mann-Whitney test.

**Fig 3:**
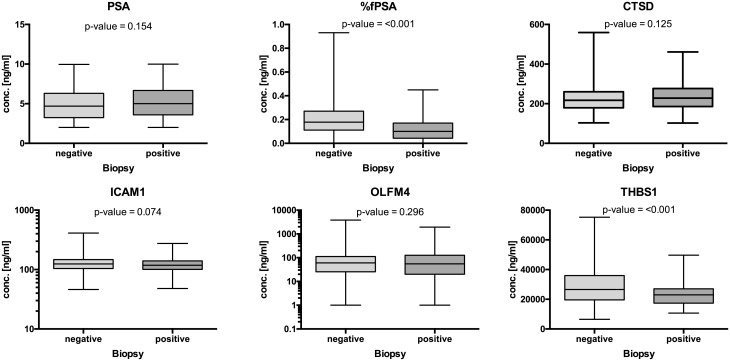
Univariate analysis of individual parameters. Box plots show the concentration of PSA, %fPSA, CTSD, ICAM1, OLFM4, and THBS1 in PCa positive and negative samples.

**Fig 4:**
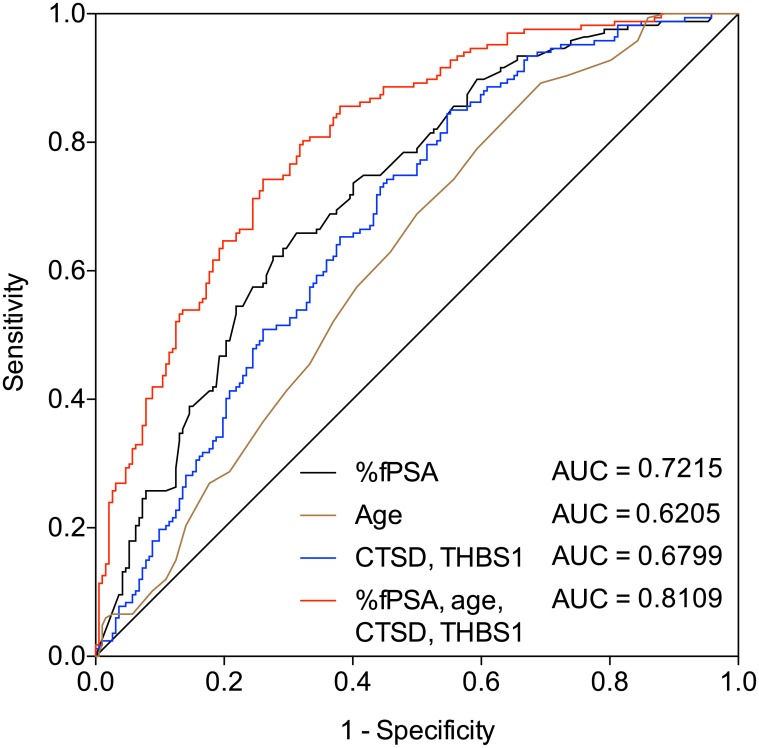
Receiver operating characteristics (ROC) analysis of different models. ROC curves depicting the accuracy in predicting PCa in 359 men who had PSA levels of 2–10 ng/ml (192 PCa negative and 167 PCa positive cases). ROC curves highlight benefits of the CTSD and THBS1 alone (blue line), %fPSA (black line), age (brown line) and the combination of %fPSA, age, CTSD and THBS1 (red line).

First, univariate analysis of the individual protein analytes was performed (Fig 3) as well as ROC analysis (Table 3, S2 Fig). Based on these analyses, %fPSA and THBS1 showed the best discrimination power between PCa negative and positive men. In a next step, the individual protein analytes as well as age were combined using logistic regression analysis. Both stepwise forward selection as well as stepwise backward elimination yielded the same model including CTSD, THBS1, %fPSA and age, respectively. This model discriminated between men with negative prostate biopsies and PCa bearing patients with an AUC = 0.8109 (*P* <0.0001; 95% CI = 0.7673–0.8545). The combination of CTSD and THBS1 resulted in an AUC = 0.6799 (*P* <0.0001; 95% CI = 0.6251–0.7346), while %fPSA alone had an AUC = 0.7215 (*P* <0.0001; 95% CI = 0.6693–0.7736) and age an AUC = 0.6205 (*P* <0.0001; 95% CI = 0.5630–0.6779) as shown in Fig 4. The combination including all four parameters was significantly better than %fPSA and age alone (p<0.001 for both contrasts). At 90% sensitivity for PCa and NPV ≥95% for high-grade cancer (Gleason score ≥7), the specificity of %fPSA, age, CTSD and THBS1 was 54%. At a more stringent sensitivity cut-off of 95% sensitivity for PCa, the specificity was 39%. In addition, decision curve analysis was performed (S3 Fig). Determining which men should be biopsied using the proposed method is superior to perform a biopsy in all men with elevated PSA or using %fPSA or age, respectively, once the threshold probability reaches about 10% or 30%, respectively, and is superior to the strategy of not performing a biopsy up to a threshold probability of about 80%.

**Table 3:** Receiver operating characteristics (ROC) of the individual analytes.

Analyte	AUC	95% CI	p-value
**PSA**	0.5436	0.4839–0.6034	0.154
**%fPSA**	0.7215	0.6693–0.7736	<0.001
**CTSD**	0.5485	0.4898–0.6071	0.105
**ICAM1**	0.5542	0.4961–0.6123	0.070
**OLFM4**	0.5297	0.4709–0.5885	0.321
**THBS1**	0.6127	0.5507–0.6748	<0.001

PSA = prostate specific antigen; %fPSA = free to total PSA ratio; CTSD = cathepsin D; ICAM1 = intercellular adhesion molecule 1; OLFM4 = olfactomedin 4; THBS1 = thrombospondin 1

## Discussion

The discovery of new biomarkers is challenging, but even more difficult is the development of a diagnostic method to substantiate clinical utility and ultimately to introduce it into clinical practice [15]. Here we have translated early research findings that identified protein biomarkers for PCa diagnosis following a targeted proteomic strategy [16] using both mouse models and patients cohorts, into an immunoassay-based diagnostic tool that can be applied into a clinical test setting.

The use of a mouse model combined with state of the art mass spectrometric analysis identified promising biomarker candidates for the diagnosis of PCa [6] after initial clinical testing. In order to overcome the analytical challenge of mass spectrometry in terms of throughput and cost, we have developed four robust and reproducible singleplex immunoassays for the individual protein biomarkers.

The Luminex technology used here provided a versatile platform to develop new immunoassays. The multiplexing technology facilitated the identification of antibody sandwich pairs by coupling several candidate capture antibodies to different microsphere sets. When mixed together, these sets allowed for the simultaneous testing of separate detection antibodies saving reagents, sample and time [9]. In addition, the reagents including antibodies and protein standards selected for the microparticle-based assays are transferable to both microtiter plate-based assays as well as potentially to random-access systems used in routine diagnostics. Given the large difference in protein concentration and thus the resulting difference in assay dilution as well as the use of different assay buffers prohibited direct multiplexing and thus the measurement was carried out in singleplex format. Nevertheless, the assays can potentially be further developed to be used in a multiplexed fashion even though immunoassay platforms with multiplexing-capabilities are rarely used today in clinical routine diagnostic.

We determined the performance of the individual assays according to standard guidelines [10]. While there might still be room for improvement for some of the assay specifications, the assays are stable and reproducible and can thus be used for evaluation in larger clinical studies.

The clinical results reported here further evaluated the role of the four glycoproteins CTSD, ICAM1, OLFM4 and THBS1, formerly identified using mass spectrometry-based measurements in early PCa diagnosis. The function of all four glycoproteins is covered by the major hallmarks of cancer development as defined by Hanahan and Weinberg [17] including apoptosis, angiogenesis and metastasis. OLFM4 was shown to be an important regulator of apoptosis in murine prostate tumor cells [18]. THBS1 was reported to be a regulator of angiogenesis in malignant and nonmalignant prostate tissue [19]. Furthermore, it was demonstrated that both CTSD and ICAM1 were promoting malignancy in the prostatic epithelium [20,21].

In this study setting, only THBS1 and %fPSA were significantly different in abundance in univariate analysis between the PCa positive and negative group, but CTSD proofed to have an additional statistically significant (*P* = 0.001) benefit when combined with THBS1, while ICAM1 and OLFM4 did not.

Both THBS1 and CTSD have been shown to be involved in PCa development [22,23]. The anti-angiogenic THBS1 suppresses the neovascularization from the existing vascular system and thus inhibits tumor growth [24,25] by directly regulating VEGF [26]. THBS1 expression was reported to inversely correlate with malignant progression [24,27]. Specifically, THBS1 immunostaining was shown to be highly positive in the benign stromal tissue and only weakly positive in the peritumoral tissue of PCa [25]. In addition, the same was shown in preclinical models as well as in patients with various types of cancer, including melanoma, breast, lung and bladder cancer [28–31]. Cathepsins in general are known to be highly expressed in various human cancers, associated with tumor metastasis [32]. CTSD was shown to be overexpressed and secreted by several types of tumor cells including lung, breast and PCa [23]. Immunohistochemical examination of CTSD was shown to reveal low levels of expression in normal prostate tissue and prominent stromal expression in malignant areas of the prostate [20]. Several studies have already suggested that CTSD acts as an essential mediator to promote malignancy of benign prostatic epithelium and therefore might be useful in differentiating benign from malign prostatic disease [33–35].

The combination of CTSD, THBS1, %fPSA and age showed clear improvement in terms of higher AUC and specificity when compared to %fPSA alone. More specifically, the results of our study showed 55% specificity at a high NPV (≥95%) for high-grade cancer (Gleason score ≥7) indicating that in clinical practice more than half of the negative biopsies could have been prevented at a low likelihood of delaying the diagnosis of significant PCa. In addition, we employed decision-analytic methods to evaluate the potential impact of the method presented here in clinical practice. Decision analysis proposes it might be an effective method in deciding whether to biopsy or not. This confirms the added value of two of four cancer-specific protein biomarkers (CTSD and THBS1) and their complementarity to the standard %fPSA test.

In 2012, a US national task force found that the risks of routine PSA screening outweigh the benefits. Recently, two new studies confirmed that the recommendation against PSA screening was associated with a change in biopsy and cancer detection rates. The studies reported that fewer early-stage PCa in general and fewer high-grade cancers in particular are being detected as fewer men are being screened [36,37]. Thus, there is an urgent need for improving the high false-positive rate of PSA for early PCa detection. Similar methods and markers for the diagnosis of PCa have been described for non-invasive testing in blood [38] and urine [39]. Two similar tests composed of kallikrein panels are available for blood-based testing. The Prostate Health Index test analyzes total, free and a precursor of PSA (p2PSA) [40] while the 4KScore includes total, free, intact PSA and human kallikrein 2 (hK2) [41]. The PCA3 test is a gene-based test in urine [42]. Similar tests exist for the detection of urine-based TMPRSS2:ERG fusion in combination with PCA3 [43] or a molecular signature of PCA3 and ERG exosomal RNA from urine [44]. Recently, a model including a combination of plasma protein biomarkers, genetic polymorphisms and clinical variables was evaluated in a large prospective population-based diagnostic study in Stockholm [45].

Larger, prospective multicenter studies are already ongoing to further validate the findings presented here. Ultimately, the method presented is intended to be used as a reflex-test if the PSA is increased, complementary to the existing %fPSA as an aid in distinguishing PCa from benign prostatic conditions. Prostate biopsy will still be required for diagnosis of PCa.

In conclusion, we provide evidence that the newly developed method presented here including the measurement of CTSD and THBS1 in combination with %fPSA and age shows improved performance in diagnosing PCa.

## Supporting information

S1 Fig Dilutional linearity experiments. The y-axis shows the analyte concentration as determined by the individual immunoassay tests using 5 serum samples for the individual immunoassay of CTSD, ICAM1, OLFM4, and THBS1.
(EPS)

S2 Fig Receiver operating characteristics (ROC) analysis of individual parameters. ROC curves depicting the accuracy in predicting positive biopsy in 359 men who underwent prostate biopsy and who had PSA levels of 2–10 ng/ml (192 controls and 167 PCa cases). (A) ROC curve of PSA (black line), %fPSA (purple line), CTSD (orange line), ICAM1 (brown line), OLFM4 (blue line), and THBS1 (red line) are shown.
(EPS)

S3 Fig Decision curve analysis showing the effect of the four models on the detection of PCa. Clinical net benefit for the models is plotted against the risk threshold at which a patient or clinician would opt for biopsy. As a comparison, the gray line represents the strategy of performing a biopsy in all men, and the black line represents the strategy of no men undergoing biopsy.
(TIF)
