# Supplementary figures and images for: Development and clinical testing of individual immunoassays for the quantification of serum glycoproteins to diagnose prostate cancer
Source: PLoS One. 2017 Aug 2;12(8):e0181557. doi: 10.1371/journal.pone.0181557 (PMC5540289; doi:10.1371/journal.pone.0181557)

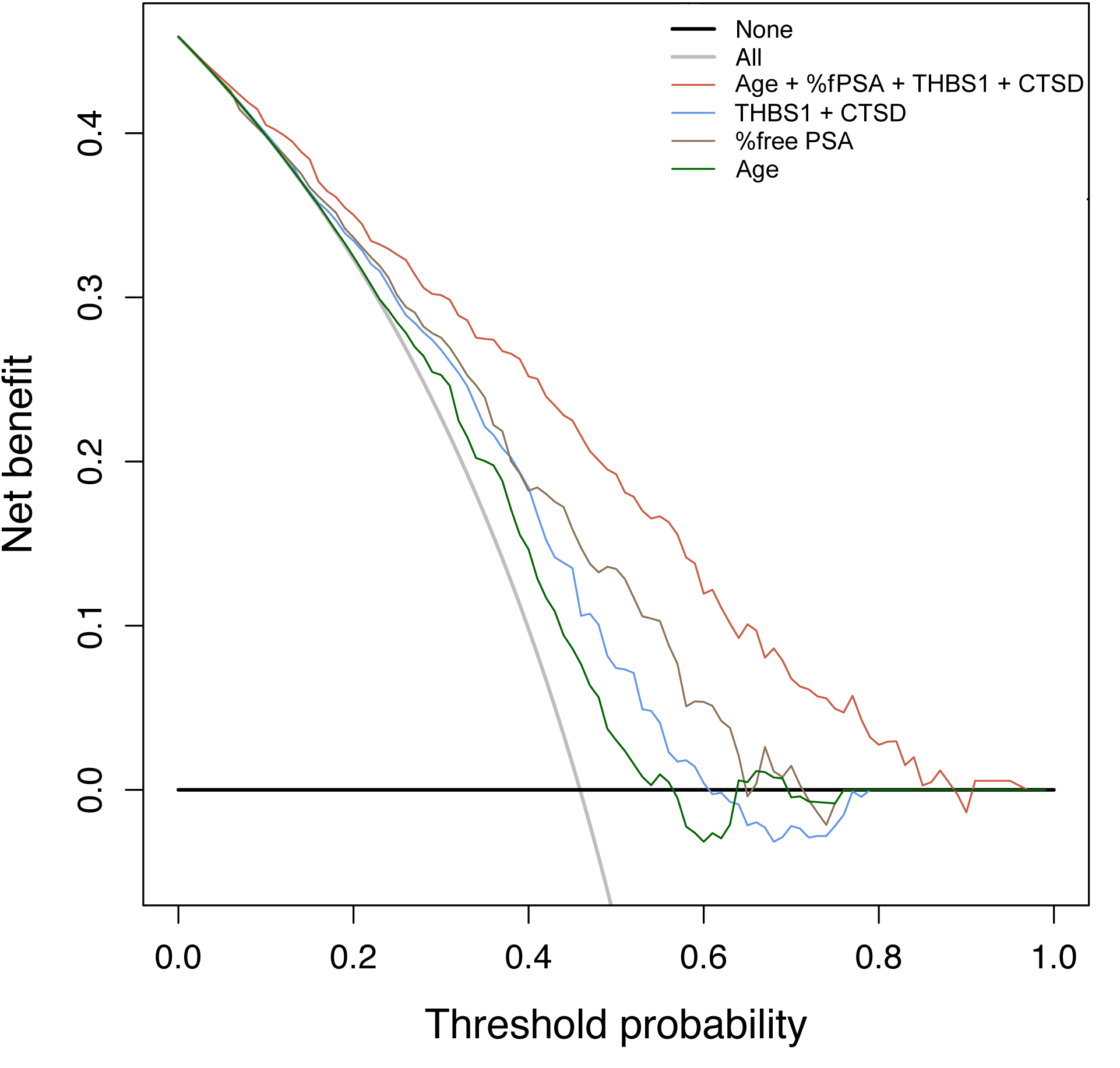

Supplement: S3 Fig — Clinical net benefit for the models is plotted against the risk threshold at which a patient or clinician would opt for biopsy. As a comparison, the gray line represents the strategy of performing a biopsy in all men, and the black line represents the strategy of no men undergoing biopsy. (TIF) [file pone.0181557.s003.tif]
